# Bluetooth Low Energy Interference Awareness Scheme and Improved Channel Selection Algorithm for Connection Robustness

**DOI:** 10.3390/s21072257

**Published:** 2021-03-24

**Authors:** Bozheng Pang, Kristof T’Jonck, Tim Claeys, Davy Pissoort, Hans Hallez, Jeroen Boydens

**Affiliations:** 1 M-Group, DistriNet, Department of Computer Science, KU Leuven Bruges Campus, 8200 Bruges, Belgium; kristof.tjonck@kuleuven.be (K.T.); hans.hallez@kuleuven.be (H.H.); jeroen.boydens@kuleuven.be (J.B.); 2 M-Group, WaveCoRE, Department of Electrical Engineering, KU Leuven Bruges Campus, 8200 Bruges, Belgium; tim.claeys@kuleuven.be (T.C.); davy.pissoort@kuleuven.be (D.P.)

**Keywords:** Bluetooth Low Energy (BLE), link layer, interference, channel selection algorithm, reliability, robustness

## Abstract

Bluetooth Low Energy (BLE) is a popular wireless communication protocol heavily used in Internet of Things applications. Nowadays, robustness is considered a key requirement in wireless communication. However, radio interference from various sources may affect the performance of BLE devices, leading to channel congestion. Therefore, there is a broadly recognized need of methodologies capable of sensing and avoiding interference. In this paper, two improvements at the data link layer for interference detection and channel selection are proposed to enhance the BLE connection robustness. This paper also presents a wide range of experimental evaluations aiming at validating the improvements and providing insights on both these improvements. Particularly, the communication performance of the BLE link layer is assessed in terms of channel usage distribution, supervision timeout ratio (STR) and packet loss rate (PLR) under different interference environments. Results from these experiments (reliability over 97% and 99% under two different harsh environments) highlight the effects of both improvements on the BLE robustness. Meanwhile, the authority of scheduling the whole mechanism is given to the link layer and even the higher application layer. This paper provides a set of solutions for BLE confronting interference in link layer.

## 1. Introduction

Wireless communication applied in Internet of Things (IoT) is reaching a significant diffusion level nowadays. The integration of embedded systems and wireless communication forms the foundation for IoT systems. With wireless communication, more applications are possible for embedded devices, ranging from daily life, such as healthcare and infotainment, to industry [1,2].

Bluetooth Low Energy (BLE) is a new wireless communication protocol introduced since Bluetooth 4.0. BLE works in the 2.4 GHz frequency band, which is known as the Industrial, Scientific and Medical (ISM) band. In this ISM band, there are diverse wireless communication protocols coexisting such as Bluetooth, Wi-Fi, ZigBee, etc. [3,4,5]. Moreover, other electronic devices make use of this ISM band such as wireless phones. Inevitably, there is unpredictable interference between previously mentioned protocols and devices, leading to conflicts between them. Therefore, performance of wireless communication is dramatically impaired, including its energy efficiency, latency amd robustness [6,7]. To avoid congestion between channels in the ISM band, BLE uses an adaptive frequency hopping (AFH) technique. The purpose of this technique is to prevent interference while keeping the communication process going [8,9].

The AFH technique is less likely to be able to cope with the increasingly complex wireless environments [3]. This further increases the possibility of a link layer transmission failure, which may lead to a lower energy efficiency, a higher transmission delay and increased latency. Although the BLE specification provides AFH with the authority to block and unblock the data channels based on their interference conditions, the BLE specification does not provide details on how and when the AFH should do that [8,9]. This results in many BLE systems, such as Zephyr RTOS and Raspberry Pi 3, not implementing any channel blocking strategies or only implementing basic channel blocking strategies which are not effective enough [10]. To correctly implement the blocking and unblocking of channels, three challenges are identified:

1. Accurate interference detection. Due to the complexity of interference in the real world, detecting this interference accurately becomes one of the main requirements for AFH. The interference can be estimated by some metrics inside BLE, such as supervision timeout (ST) and packet loss (PL). However, which ones to be used and how to use them are key challenges.
2. Continuous channel monitoring. Due to the lack of predictability of interference, another requirement for AFH is to monitor the interference and channel conditions continuously. Neither a periodic detection nor a random sampling detection seems a logical choice for continuously changing interference. Thus, a continuous monitoring for the connection is considered as the best choice, even under some special situations, such as channel map blacklisting or whitelisting procedures.
3. Compatibility. The integration of the interference detection process into the AFH is also challenging. We have to pay attention to whether there is any conflict between them. Some further improvements or even changes are needed if the conflict is irreconcilable.

Keeping those challenges in mind, this paper proposes a comprehensive strategy on how to improve the connection robustness under interference for BLE devices. We conclude the main contributions of our work as follows:

1. Interference awareness. An interference awareness scheme is proposed, based on the packet status of a BLE connection. In the BLE connection, multiple aspects of each packet transmitted and received are monitored so that we can detect the interference as soon as possible.
2. Interference avoidance. Some improvements for the channel selection process which rely more on probability are proposed. The information of interference is collected in the algorithm and helps the BLE to choose a data channel based on probability.
3. Validation experiments. To validate both the interference awareness process and the avoidance process, they are further proved by testing them with experiments under various environments. Experimental results are discussed in detail to show the performance improvement.

The rest of this paper is organized as follows. Related work is presented in Section 2. In Section 3, the background on BLE connections is introduced. Section 4 discusses robustness and some essential metrics of the link layer in BLE. Section 5 illustrates the interference awareness scheme (IAS) and improved channel selection algorithm (CSA). In Section 6, experiments and related results are described and provided. In Section 7, conclusions with final considerations and remarks are drawn.

## 2. Related Work

From a wider perspective, different wireless communication protocols use different frequency hopping algorithms. Many researchers have investigated their coexistence and performance. To improve different frequency hopping algorithms, various theoretical approaches, algorithms and experiments are proposed in [11,12,13,14,15,16,17,18,19]. For instance, in [11], the authors introduced a data channel selection scheme with proactive hand-off in a cognitive radio network. Their algorithm improves the communication performance through the best channel selection. The principle of the algorithm is reducing the channel switching number, thus minimizing interference due to primary user activities. Riyadh A. Abbas et al. [12] studied a novel randomization algorithm for the channel selection process of the Weightless-N system. Their new randomization algorithm is based on a uniform randomization distribution which is quite similar to the pseudo-random number in the BLE. The results of their algorithm provide a better system performance and lower probability of collision. Ali et al. [13] proposed a M/M/1 queuing model for spectrum handoff in cognitive radio networks and a shortest queuing selection model to the backup channel after the handoff process occurs. Their results show the quality of service for the secondary user can be improved by their methodology. Moreover, in [14], it is mentioned that channel selection is one of the most important issues in cognitive radio networks. The authors discussed channel and corresponding rate selection techniques and gave the successful transmission probability for a selected channel. In [15], an extension to the predictive channel selection algorithm framework and its generalization for heavy-tailed primary users of time distribution are introduced. The authors implemented their channel selection framework in a wireless testbed and comprehensively evaluated the performance. However, their focus was on channel switch and energy consumption. To solve interference issues, Carhacioglu et al. [16] proposed a cooperative coexistence solution for BLE and time slotted channel hopping networks in which joint time-slot and channel hopping synchronization are performed. The authors evaluated the performance of their proposed mechanism using experiments with real wireless devices, and they mainly paid attention to packet error rate. Yuki Nishio et al. [17] constructed the optimal channel selection rate of slave based on theoretical analysis; Kavita Mathur et al. [18] studied how to improve the throughput by using dynamic channel selection in 2.4 GHz band of IEEE 802.11 WLAN; and Mahesh Y. Sumthane et al. [19] performed sets of experiments to detect interference with the help of IEEE 802.15.4 wireless sensor networks hardware kit and also developed an algorithm to control the effect of Wi-Fi interference on IEEE 802.15.4 wireless sensor network. Comparing with all the papers above, this paper aims at improving the BLE channel selection algorithm so that the BLE connection reliability can be ensured while facing strong interference.

R. Natarajan et al. [3] investigated the coexistence of popular IoT wireless communication protocols, e.g., IEEE 802.15.4, BLE and IEEE 802.11. All of them were studied and compared in detail to illustrate interference in the ISM band. For BLE, the effect of the AFH and re-transmission under interference was also illustrated. The results showed that AFH and retransmission could improve the reliability of the BLE connection, but with a cost of latency. However, even with the AFH and retransmission, the BLE connection is not reliable in some cases, such as at short interference intervals. The authors suggested that further improving the reliability of BLE is possible by adding some extra techniques, such as Carrier Sense Multiple Access (CSMA). A. Ancans et al. [20] studied the BLE throughput under Wi-Fi interference and other BLE devices. The parameters, such as the BLE connection interval (CI) and PHY mode, which are mainly used to adjust the throughput and communication range, were considered in their work. Their results originated from experiments based on development boards, providing the conclusion that Wi-Fi activity reduces BLE throughput by approximately 30% regardless of the CI. However, the researchers only looked at performance analysis without any improvement for BLE. In contrast to these papers, we aim to improve the BLE performance under interference. M. O. A. Kalaa and H. H. Refai [7] investigated the channel usage probability of BLE and communication collision probability for multiple BLE pairs. Clear probability distributions for both were analyzed mathematically. Their results prove that there is an uneven distribution inside the BLE channel selection. Meanwhile, multiple BLE devices would increase the possibility of communication collision. However, they did not provide any solutions for those phenomena. This paper aims to propose methods that are better at solving communication collision.

M. C. H. Chek and Y. K. Kwok [21] studied the coexistence between Bluetooth and Wi-Fi (IEEE 802.11b) and proposed a novel approach to avoid interference, called ISOAFH. However, they only paid attention to Bluetooth and IEEE802.11b and only simulated their AFH algorithms. W. Lee and H. Kim [22] provided a strategy called BluS to help Bluetooth devices avoid interference. BluS is able to sense the environment continuously, without constructing a Bluetooth connection. BluS can also estimate channels’ qualities of different standards accurately using the 2.4 GHz ISM band. Furthermore, their work was proven as energy efficient, and it would not degrade the quality of data communication. This research provided some deep insights about interference avoidance. However, they only concentrated on Bluetooth, instead of BLE, which are largely different from one another. M. Spork et al. [23,24] researched on how to improve timeliness and reliability for the BLE connection under interference. The methodology they mainly concentrated on was to adjust various parameters during communication, for example, the connection interval and PHY mode. They also proposed a methodology about detecting, blacklisting and whitelisting BLE data channels, which may keep the BLE communication reliability high, even 98.6% on average, but their methodology has some drawbacks which are solved in this paper. Their research shows great applicability and seems a positive supplement to BLE but lacks flexibility for link layer implementation.

## 3. Background on BLE Connections

### 3.1. BLE Communication Process

**Figure 1:**
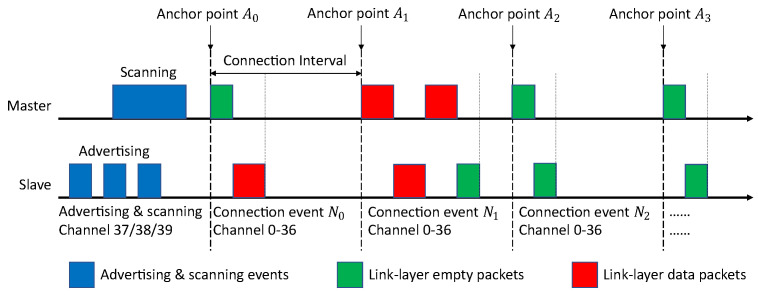
The BLE connection between a master and a slave.

BLE operates in the license-free 2.4 GHz ISM band. In BLE, this ISM band is divided into 40 communication channels, having a bandwidth of 2 MHz each. Those 40 channels include 3 advertising channels and 37 connection channels. The two different types of BLE channels are used for different BLE communication modes. BLE provides two communication modes: a connection-less mode and a connection-oriented mode. The connection-less mode uses three advertising channels (37–39) to broadcast advertising packets. This advertising packet is needed to set up the connection-oriented mode. The connection-oriented mode uses the other 37 connection channels (0–36) to exchange data. During connection-oriented mode, one BLE device acts as the master and the other one acts as the slave. Bidirectional data transfers only happens in connection oriented mode [8,9]. Each connection channel is divided into multiple CIs, as shown in Figure 1. Note that this CI is the time limit of connection events and a connection event ends with the last packet being sent from the slave.

Each connection event occupies an uncertain period inside a connection interval, depending on the communication demand. The connection event lasts a time period, limited by the CI but decided by the application. A CI is counted as the time duration between the start of two consecutive connection events. The start of a connection event is an anchor point. It is not necessary for a connection event to fill the whole connection interval. During each CI, the master and the slave exchange link-layer packets. Link-layer packets can be divided into two types: data packets and empty packets. Data packets carry application data, while empty packets carry no payload but only some necessary information, such as a header. For instance, the more data (MD) bit in the header of the packet is used to indicate that the device has more data to send. These empty packets are used to keep the BLE connection alive or to confirm the end of a connection event. Every connection event starts from the master and ends at the slave. The last link-layer packet in all the connection events should be sent from the slave. If no data need to be sent in a CI, only one pair of mandatory empty packets will be exchanged, keeping the connection alive. After exchanging a pair of empty packets, the BLE radio is turned off until the beginning of the next connection event.

As an example, shown in Figure 1, the slave device is responsible for advertising, and the master scans for advertising packets on the advertising channels. Using this protocol, they will be able to set up a connection. The master then starts the connection event, N_0_, by sending an empty packet to the slave. The slave in turn responds with a data packet, including the application data. After which, the connection enters event N_1_, and the master and slave both send application data. Connection event N_1_ ends with an empty packet sent from the slave, as an acknowledgment. During connection event N_2_, neither the master nor the slave has application data to send. Thus, they only exchange mandatory empty packets to keep their link or connection alive. Each of these three connection events uses a random channel from connection channels 0–36.

### 3.2. Anchor Point

As shown in Figure 1, the start of a connection event is called an anchor point. An anchor point is used to synchronize the master and slave. Due to clock inaccuracies, a small difference could exist between the master and slave. Therefore, the slave device shall re-synchronize to the master’s anchor point by listening to the master. The slave will use the last anchor point and an offset to calculate its next anchor point. It will start to listen to the master at that point in time. If a slave device receives a packet from the master, the slave shall update its anchor point time. Note that a cyclic redundancy check (CRC) mismatch only denotes packet errors, does not impact the anchor point synchronization. A connection event or interval starts at this point. Since the anchor point follows the master’s clock all the time, it is always the master who transmits the first connection channel protocol data unit (PDU), in the connection interval or event, to the slave [8,9].

### 3.3. Acknowledgment

In the BLE link layer, each connection event may consist of one or several pairs of transmission packets. To make sure all the packets are sent and received in sequence, BLE uses the sequence number (SN) and the next expected sequence number (NESN). Both SN and NESN contribute to the BLE acknowledgment (ACK). Both of them occupy one bit in the header of link layer packet. Both the SN and the NESN are used in a packet sent either by a master or a slave. The SN is used by the receiver to verify the current packet. The NESN is designed to acknowledge the receiver about the packet expected by the sender for their next transmission. Only when both SN and NESN match, a transmission packet is considered as successfully acknowledged. In BLE, when the SN is not equal to the NESN, they are considered as correct. Then, the master and the slave may keep exchanging data. Otherwise, a retransmission is started which only stops when a correct ACK is received or due to a timeout event [8,9].

### 3.4. AFH and CSAs

Each connection event occupies a connection channel for the duration of one CI. However, it is not necessary for the connection events to fill the full CI. Each connection event happens in a pseudo-random selected connection channel. AFH assists the BLE devices to avoid interference while keeping the connection between the master and the slave. Channel selection algorithms are algorithms inside AFH to decide the next connection channel for both the master and the slave. Currently, the BLE standard describes two types of CSAs: CSA #1 and CSA #2. If both master and slave support BLE version 5.0 or higher, they use CSA #2 by default. Otherwise, CSA #1 will be used [8,9].

Since all the BLE channels are located in the ISM band, the BLE communication will suffer from interference of other wireless technologies, for example, Wi-Fi, Bluetooth, ZigBee, etc. The PL or even link loss may happen due to interference, thus affecting the reliability of the BLE communication [8,25].

Each CSA uses the same pseudo-random number generator sharing the same parameters, so the master and the slave are able to hop to the same channel for every connection event. By continuously hopping to another channel for each new connection event, the BLE devices are able to avoid interference from the last connection channel, if any. To further strengthen the interference avoidance, a channel map mechanism is used in both CSAs. A channel map is created which contains unblocked channels and blocked channels that have a poor link quality. The quality of all the connection channels is represented by the channel map. An unblocked channel is shown as a “1” bit. A blocked one is shown as a “0” bit. The channel map update procedure is actually a procedure to update the channel map for both master and slave. This procedure is allowed at connection status and can only be initiated by the master. It means the BLE devices share the same channel map updated by the master. The BLE devices can not use blocked channels until they are unblocked again. The channel map sets all channels available by default at the beginning of a connection [8,9]. The channel map may be updated after entering the connection-oriented communication. The master controller can update the channel map without being requested to by the host. The host refers to the upper layers of the Bluetooth protocol stack, between link layer and application layer.

**Figure 2:**
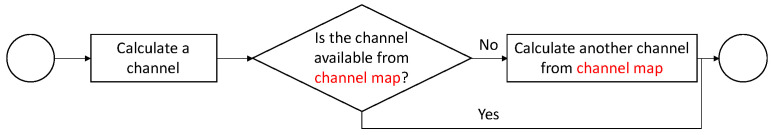
The basic logic for both CSAs in BLE.

The basic logic for both CSAs, shown in Figure 2, starts with calculating a channel index, which should be in the range of 0–36. Then, both algorithms judge the calculated channel index based on the channel map. If the channel map shows the calculated channel index is available, the channel index is used for the next connection event or interval. Otherwise, both algorithms ignore the calculated channel and calculate another one, based on the availability from the channel map. However, the BLE specification does not provide any methodology to obtain the availability from environments.

An updated channel map could improve the robustness of BLE communication. However, most BLE devices, in the market now, do not block channels in the channel map. For instance Zephyr RTOS does not update the channel map [8,10]. There are several reasons for this. For example, the BLE specification does not indicate when and how the BLE devices should detect the environment or interference. In addition, the BLE specification does not specify how frequently the BLE devices should update the channel map. Moreover, after blocking a channel, deciding when a channel can be unblocked is another challenge. All of these questions are left to developers or vendors. Until now, there is no standard methodology to cope with aforementioned challenges. Note that this paper is based on the BLE specification version 5.0 and 5.2. Even the newest BLE specification, version 5.2, does not solve the challenges above. The BLE specification version 5.2 introduces the concept of subevents into CSA #2 [8]. The subevents further divide an interval into smaller time slots, and each subevent occupies a different data channel. However, this is out of the scope of this paper.

### 3.5. Current Issues and Challenges

As mentioned above, most BLE devices do not update their channel maps due to the BLE specification not providing a compete scheme for updating these maps. The motivation behind this is the difficulty to optimize the different parameters such as energy efficiency, latency, robustness, etc. For example, on the one hand, the master senses the environment and updates the channel map frequently, the energy consumption will increase. On the other hand, if the master hardly updates the channel map, there is a higher possibility for BLE encountering interference, leading to latency and robustness issues due to PL or errors. Besides this, BLE devices, under different interference environments or with different applications, should have different frequencies to detect interference and update their channel maps accordingly. For instance, a movable BLE device may face different interference with changing environments, which necessitates sensing the environment and dynamically updating the channel map, whereas, in practice, there is no IAS implemented in BLE stack as of now [8,9].

In [24], the authors proposed a methodology to detect bad channels with PL at runtime by monitoring the packet delivery ratio. However, as discussed in Section 4.2, it is not possible to deduce information about ST and CRC by monitoring packet delivery ratio or PLR alone. More accurate information of a packet can be obtained by monitoring multiple aspects like ST and PL, thus interference could be detected more accurately.

According to the description above and the BLE specification, both CSAs from BLE are based on the channel map, determining the availability of channels for the next connection event. However, the electromagnetic environment changes all the time and depends on multiple aspects, such as location, transmission power and antenna direction [26]. No matter when the channel map is updated by the master, it is only representative for that moment and that specific location. Hence, it is risky to choose a channel based on the channel map. However, it is also impossible to sense the environment and update the channel map all the time continuously.

A simple combination of an interference detection scheme and BLE CSAs would result in a continuously decreasing number of available channels because theoretically both CSAs only rely on the channel map in the link layer and would not give the interference detection scheme any chance to unblock a channel. To reliably re-enable a blocked channel, the interference detection scheme has to test the channel by one connection event at least. However, based on the logic from both CSAs, if a channel is blocked in the channel map, the master and the slave will no longer use that channel. Consequently, there is no opportunity for the interference detection scheme to test and re-enable the blocked channels. This is the reason why a new CSA is needed.

To solve the issues above, Spörk et al. [24] whitelisted all the blocked channels when the number of active channels drops below a minimum number defined by themselves, so that the BLE can probe all the channels again to measure the interference. To ensure a reliable data exchange during whitelisting, Spörk et al. [24] adapted the BLE CI to a shorter value before re-enabling the data channels. In addition, to ensure the most accurate estimation of each channel’s link quality, Spörk et al. [24] proposed to disable the channel blacklisting process temporarily. By all these steps, the blocked channels can be re-enabled again, and an average packet delivery ratio of 98.6% can be reached in a static interference environment. However, there are a few issues in the methodology. First, the minimum number of channels is dependent on the situation and has to be set beforehand, and whitelisting all the blocked channels is risky under an interference environment, leading to packet collision. Second, there is no proof showing that adapting the BLE CI to a shorter value can ensure a reliable data exchange. Third, disabling the blacklisting process makes the channel map not timely update anymore, which may further lead to a difference between the later updated channel map and the environment.

## 4. BLE Link Layer Robustness

To illustrate the robustness of the link layer in BLE, the basic process, accompanied with theoretical analysis, is shown here. First, two link-layer parameters, STR and PLR, are listed in Section 4.1. Then, a theoretical analysis is carried out in Section 4.2.

### 4.1. Link Layer Parameters

#### 4.1.1. Supervision Timeout Ratio

Due to multiple reasons, e.g., a device moving out of range, encountering severe interference or power failure conditions, a slave may not hear from its accompanying master at the calculated anchor point. However, the slave still turns on its radio to wait for the first transmitted packet from the master. After a certain period of a transmit window size, the slave turns off its radio until the next calculated anchor point. Once this happens, it increments a fail counter of anchor point synchronization. Due to this failure, the BLE devices are not able to exchange any data in the current CI. Thus, this event is called an ST occurring in the link layer [27]. Note that, in this paper, only the situation of encountering severe interference is considered.

As mentioned above, multiple reasons may cause anchor point synchronization failures. On top of that, malformed packets (with CRC errors) are also counted as ST events. Following the specification, the timeout shall be a multiple of 10 ms in the range of 100 ms to 32.0 s. After that, BLE considers the link to be lost [8,9]. However, in this paper, we use the ST event in another way. We define the ST event as the ST timer starts to count, since we consider once the timer starts to count, it means something unexpected happens (PL, packet error, etc.).

Here, the supervision timeout ratio or STR is defined as:

(1) $$\mathrm{STR}=\frac{\#ST\_events(sender)}{\#TX(sender\to receiver)},$$

where the #ST_events(sender) is the number of ST timer starting events on the sender side and the #TX(sender→receiver) is the total number of packets sent during the whole communication. Both numbers are calculated from the existing BLE link layer functions. Based on these two calculated numbers, STR is calculated. Note that STR can be calculated at both sides of the BLE connection, but only the one calculated from the master side is used. The channel map can only be updated from the master, by sending a protocol data unit containing relevant information to the slave. The protocol data unit also states when the master and the slave should start to use the new channel map.

STR provides information about both the anchor point synchronization status and the packet CRC status. It should be calculated first at the beginning of a connection interval or event and also after each received packet.

#### 4.1.2. Packet Loss Rate

To judge the robustness of a communication process, PLR is mostly used. For various reasons, packets sent may be lost or incorrect, resulting in no ACK or a wrong ACK. In this case, the packet is classified as a failed transmission. A re-transmission process is required until the sender receives a valid ACK. In BLE, ACK is achieved by SN and NESN as mentioned before. As long as SN is not equal to NESN, the packet is considered as acknowledged successfully.

The packet loss rate or PLR can be calculated as:

(2) $$\mathrm{PLR}=1-\frac{\#ACK(receiver\to sender)}{\#TX(sender\to receiver)},$$

where #ACK(receiver→sender) is the number of received packets with valid ACKs and #TX(sender→receiver) is the same as above, the number of packets sent. Similar to STR, both numbers are extracted from the existing BLE link layer functions. From these two extracted numbers, the PLR is evaluated.

The PLR is able to show packet transmission status in the BLE link layer. It is therefore calculated after each received packet so that the connection is continuously monitored.

### 4.2. Case Analysis

A deep understanding of STR and PLR is needed to decide how they are used in our experiments. Some theoretical analysis is necessary to see how they perform in BLE connection status.

#### 4.2.1. Case 1

**Figure 3:**
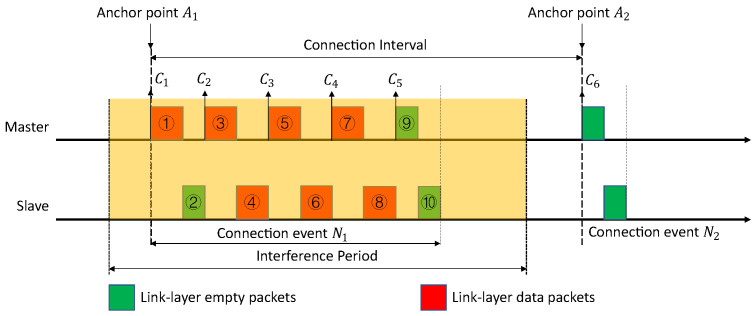
The connection case 1 under the interference.

As shown in Figure 3, the whole connection event N_1_ is under a strong interference. The CI starts from anchor point A_1_ and ends with anchor point A_2_. There are ten packets inside this connection event, with five packets from the master and five from the slave. In this scenario, all of them are under interference, including the anchor point A_1_. Multiple possibilities could happen to the packets under an uncertain noise level. Some examples are given below.

1. Assuming that packet ① is received successfully by the slave. At anchor point A_1_, the master sends the initial packet of connection event N_1_ to its slave. Since this packet ① is received successfully by the slave, the anchor point A_1_ is synchronized between the master and the slave. After packet ①, the slave will send packet ② back to the master. If the master receives this packet ② successfully, the STR and PLR can be calculated at point C_2_ according to the packets ① and ②.
2. Assuming that packet ① is not received successfully by the slave. At anchor point A_1_, the master sends the initial packet of the connection event N_1_ to the slave. Since the packet ① is not received successfully by the slave, the anchor point A_1_ is therefore not synchronized. That leads to a failure of all the following packets, and packet ① is the only one left in the whole CI. In this situation, the STR rises at anchor point A_1_ or point C_1_. However, PLR cannot be calculated until the end of this CI, at anchor point A_2_ or point C_6_.
3. Assuming that both packets ① and ② are transmitted successfully, and packet ③ is received by the slave without CRC or ACK errors. The slave starts to transmit packet ④. If this packet ④ is received by the master with CRC or ACK errors, the master will ask for a re-transmission from the slave. In this situation, both the STR and the PLR can be calculated at point C_3_. However, depending on the type of errors, only one of the results or both can tell the status of packet ③ and ④. Please note that, in BLE, there could be ACK errors with a correct CRC.
4. Assuming that all the packets in the connection event N_1_ are successfully transmitted but partially correct. The connection event N_1_ ends with packet ⑩. The STR and the PLR are able to monitor every pair of data packets sent between the master and the slave. The STR is calculated from point C_1_ to C_5_. The PLR is calculated from C_2_ to C_6_. With both STR and PLR, the whole CI, from C_1_ to C_6_, is under monitoring.

Thus, under different situations, STR and PLR perform differently and have different interpretations. Basically, STR pays more attention to the beginning of every CI, and, meanwhile, it is able to declare CRC errors for each received packet. The PLR could provide insight in the ACK status of each received packet, and it is able to monitor the PL status of all the packets. To have a complete understanding of the whole CI, both STR and PLR are necessary.

#### 4.2.2. Case 2

**Figure 4:**
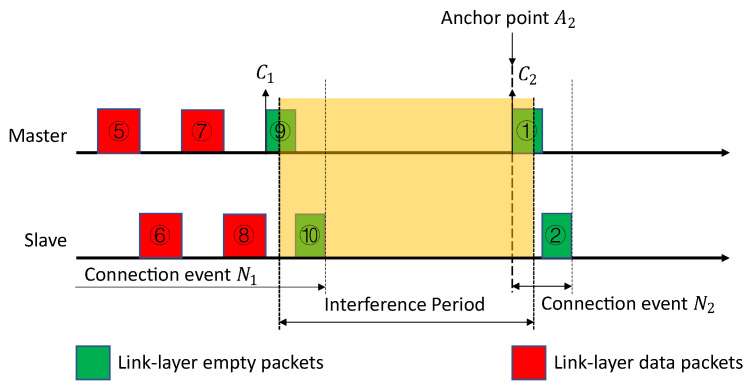
The connection case 2 under the interference.

As shown in Figure 4, a period of strong interference happens from the mid of the packet ⑨ to the mid of the packet ①. Packet ⑩ is under the interference. Again, there may be various situations happening to this packet, and some examples are shown below.

1. Assuming that packet ⑨ in connection event N_1_ is transmitted successfully and correctly. It does not matter if the packet ⑩ is transmitted successfully or not, at point C_2_, the PLR for the packets ⑨ and ⑩ in the connection event N_1_ is calculated. It can be used to show the transmission status and interference for the packets ⑨ and ⑩.
2. Assuming that packet ① in connection event N_2_ is received successfully by the slave. The anchor point A_2_ is synchronized between the master and the slave. At point C_2_, the STR can be calculated for packet ① in the connection event N_2_. It can be used to show the anchor point synchronization status and interference for the packet ①. Under this situation, the PLR is not able to tell anything about the interference happening.

The ST event is counted at the beginning of each packet no matter the BLE device receives it or not, while the PL event can only be counted at the end of each received packet. Thus, the ST event is always faster than the PL event to show the channel status. Especially when looking at the anchor point, a PL event at that point does not trigger a change to the PLR immediately (because the PL event is only counted after receiving a packet); however, an ST event triggers a change to the STR immediately (because the ST event is checked no matter the packet is received or not). Thus, looking at the anchor point, we have an STR already, but we have no PLR. Thus, we consider the ST event is a quicker indication of the current connection interval for its channel status.

The two examples above show that the interference information about two continuous connection intervals can be roughly extracted at the anchor point. From this point of view, the PL event can be seen as an indication of the channel status for the previous connection interval. The ST event can be considered as an indication for the current connection interval, since it is able to tell the channel status close to the anchor point.

#### 4.2.3. Analytical Results

To monitor the communication process continuously and accurately, both the ST event and the PL event should be used for each connection interval. Only with both of them is interference detection able to cover all the cases that could happen during the whole communication. Meanwhile, they can guarantee the timeliness of interference detection. To show the robustness or reliability for the entire communication process, the STR and the PLR are calculated cumulatively.

From a systematic perspective, the BLE connection is a repetition of many CIs and the only difference is the CEs inside. Our case analysis includes the situations that the interference happens within one CI and over two CIs, but it can be extended to multiple CIs which means the whole connection.

## 5. IAS and Improved CSA

### 5.1. Interference Awareness Scheme (IAS)

In this section, we introduce a novel BLE interference awareness scheme considering all the factors discussed above. Both the ST event and PL event are used to increase the speed of sensing the environment and provide more accurate information about the interference. The direct results of interference are PL or ST, thus both the ST event and PL event are used to achieve interference awareness. This decision is drawn from our case analysis because both the ST event and PL event show their own advantages and functionalities. For instance, the ST event has an advantage to tell the channel status at its anchor point but the PL event concludes the channel status after each packet. Moreover, a function of the ST event is to check the CRC error and the PLR checks the ACK status.

The basic logic of the IAS is illustrated in Algorithm 1. A CI or event starts with a channel calculated by one of the CSAs. In this CI, both the ST event and PL event are supervised by the master. To make the interference detection sensitive, as long as either of these is detected, the used channel will be blocked because, theoretically, there will be no ST event or PL event if there is no interference. Meanwhile, the master updates the channel map for both itself and the slave, by sending a protocol data unit including relevant information. After each connection event, the channel which causes any ST event or PL event is blocked for both the master and the slave. If there is no ST event or PL event, the channel used will be unblocked. Then, the master will check if all the channels will be available after unblocking the channel. If yes, the master shall also update the channel map by sending the new channel map to the slave. This is to ensure the energy efficiency for BLE devices. After all, updating the channel map multiple times just because of unblocking a channel is not reasonable.
**Algorithm 1:** IAS
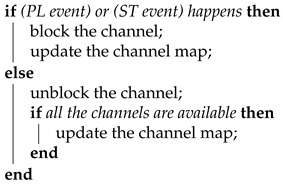


A logical conflict between IAS and CSAs from BLE is that the blocked channels can no longer be used. This conflict results in no possibility for the channels to be unblocked. Theoretically, in an interference-rich environment, the IAS will run out of channels as it blocks channels with sporadic PL events or ST events too aggressively. In [24], the authors solved this issue by setting a threshold for the packet delivery ratio and a minimum number for the available channels, as 90% and 10, respectively. Setting a specific threshold for the packet delivery ratio is feasible for static environments, but a dynamic environment cannot be managed the same way. In addition, a minimum number for the available channels will lead to a loop of blacklisting and whitelisting frequently, which will cause more power consumption and a higher PLR or STR. This issue is addressed by our improved CSA and discussed below.

Note that the channel map updating may take at least six CIs, as mentioned in the BLE specification. This may lead to inevitable delays during the updating process.

If neither an ST event nor a PL event happens and if the master finds no available channels, then there will be no channel map updating. This makes the IAS more energy efficient. Since the used channel is unblocked all the time, it is not reasonable or energy efficient to update the channel map multiple times.

### 5.2. Improved CSA

**Figure 5:**
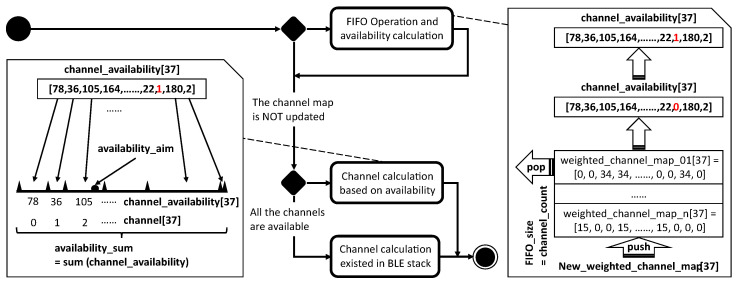
The main logic for the improved CSA.

Based on all the challenges above, the improved CSA is shown in detail in Algorithm 2 and graphically in Figure 5. First, the general principle is explained using the graphical representation. The steps in the improved CSA follow the flow chart in the graph, and they begin with a conditional decision. If the channel map is updated, the flow goes into the FIFO operation and availability calculation. The detailed steps inside are shown on the right. The FIFO is used to store the *weighted_channel_maps*, which are calculated from the updated channel maps between the master and slave. This FIFO helps BLE avoid the risk of relying on only one channel map and makes the channel selection more based on probability. The *weighted_channel_map* combines the channel map with a weight which gives the information of current interference level. The size of the FIFO is represented with *FIFO_size* which equals to the number of available channels in the last updated channel map. The *FIFO_size* follows the number of available channels to help BLE devices better adapt to the environment. The *channel_availability* is calculated by summing up each column inside the FIFO, leading to the availability that shows the probability level of having no interference at a specific channel. The higher is the availability, the lower is the probability level of having interference. After that, we check the *channel_availability* and change all the zeros into ones, to ensure a tiny chance for those channels being selected later. Hence, there is no real blacklisting in this improved CSA.
**Algorithm 2:** Improved CSA
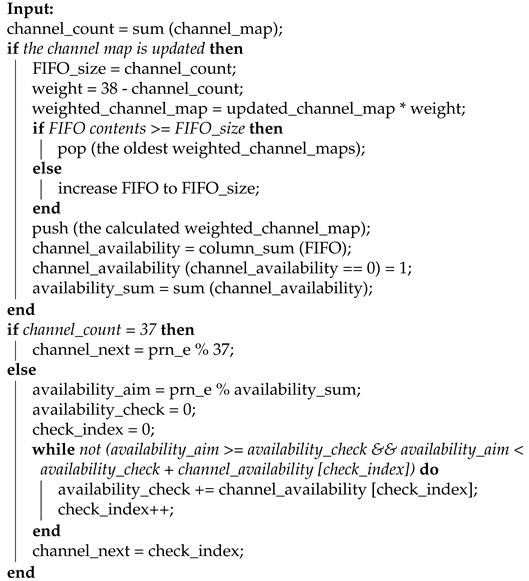


After the first conditional decision, the improved CSA meets the second one. If not all channels are available based on the channel map, the flow goes into the channel calculation based on availability. The detailed steps inside are shown on the left. To use the *channel_availability*, it is then represented by a line. The length of this line is equal to the *availability_sum* which is the sum of all *channel_availability*. The availability from *channel_availability* is aligned on the line sequentially according to the 37 channel numbers. Then, a random point named the *availability_aim* is chosen on the line. The improved CSA checks in which availability area the *availability_aim* locates, and thus the channel is found. Naturally, the larger is the availability of a channel, the higher is the chance it will be picked. For instance, in Figure 5, the *availability_aim* point locates in the availability area 105, thus channel 2 is picked correspondingly.

Algorithm 2 illustrates the details inside the improved CSA. It mainly consists of two conditional decisions (*if* statements). The first statement is only executed when there is a new channel map available. It calculates the availability for all the channels and updates the FIFO used to store weighted channel maps. The second statement is executed in every CI, because each CI needs a new channel to hop to. This is also what needs to be done based on the BLE specification. This statement calculates the channel for the next CI. The variable called *channel_count* is put in front of these two *if* statements and is used to represent the number of available channels from the channel map. By summing up all the bits inside the channel map, the *channel_count* is obtained.

The first step in the first *if* statement is to initialize the necessary variables, such as *FIFO_size* and *weight*. A *weighted_channel_map* is then calculated based on the *updated_channel_map* and *weight*. Since the *FIFO_size* can change between every updated channel map, the *FIFO_size* has to be adapted accordingly. The *FIFO_size* changes because a smaller size adjusts the availability inside faster, thus adapts the improved CSA to the environment faster, and vice versa. If the FIFO contents are larger or equal to the new *FIFO_size*, all *weighted_channel_maps* older than the new *FIFO_size* − 1 are popped because they may slow down the adaption speed. If the new *FIFO_size* is larger, the size is increased accordingly and no *weighted_channel_maps* are popped. In both cases, the latest *weighted_channel_map* will be pushed into the FIFO. By summing up a column in the FIFO, a channel availability is produced per channel. The *channel_availability* contains the availability for all the channels. If the availability is calculated as zero, it will be set to one, so that a tiny chance is still kept for those channels under strong interference to eventually be used again if the interference is gone. The *availability_sum* is the sum of all the availability in the *channel_availability*, which will be used in the next *if* statement.

In the second *if* statement, for every CI, a channel is calculated for the next CI. At first, when all channels are available, the next channel is simply chosen randomly leading to uniform distribution of channel usage when no interference is present. The *prn_e* is a 16-bit pseudo-random number defined in the BLE specification [8,9]. If not all channels are available, the availability from the first block is used to calculate the *channel_next*. First, the *availability_aim*, *availability_check* and *check_index* are initialized. The *availability_aim* is imagined as a random point on a line with a length of *availability_sum* which is divided into 37 different shorter lines to represent the availability of all the 37 channels. The *availability_check* is used to look for the area where the *availability_aim* point locates. The *check_index* indicates the channel that is calculated and chosen. The while loop checks and finds the *channel_availability* which includes the *availability_aim* point and gives the corresponding value of *check_index* to *channel_next*. With all the steps above, the channel to be used for the next CI is calculated and stored in *channel_next*.

## 6. Experiments and Results

### 6.1. Experimental Setups

To show the impact of the IAS combined with the improved CSA, they have to be tested under different EM environments. Two specific environments are selected, a controlled interference environment and an uncontrolled one, which are introduced in detail below. The BLE connection is built with two nRF52840 DK [28]. Both devices use their PCB antennas for communication.

In both the controlled and uncontrolled environments, the master initiates a BLE connection with the slave with a CI of 50 ms. To accelerate the experiment, the CI is updated to 7.5 ms—the minimum CI allowed in BLE—after several connection events. In this case, our experiments can run fast, for example, 10,000 (100,000) connection intervals only cost around 75 s (12.5 min). The connection latency is set to 0 by default and the ST is 4 s. Some other parameters are maximum transmission unit and PHY mode which are 23 bytes and LE 1M PHY mode, respectively. Table 1 presents the parameters used for our test bed.

Since most of the experiments are in the link layer, an open-source real-time operating system (RTOS), Zephyr RTOS [10], is used in the development boards. It supports version 5 of BLE, contains both BLE specified CSAs and gives the possibility to create and test the proposed IAS and CSA, while most stacks are proprietary, making them hard to adjust in lower layers. In the link layer, one can control and monitor both CSAs. The STR and PLR are extracted for all the received packets one by one. The results of the STR and PLR are logged in the link layer on the master side.

#### 6.1.1. Controlled Environment

**Figure 6:**
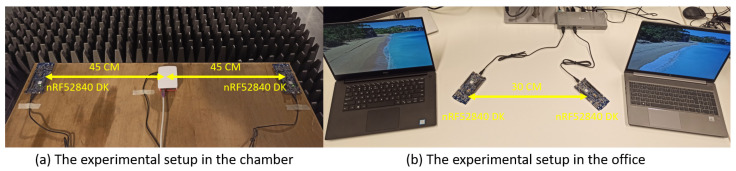
The experimental setups in the chamber and the office.

To create a controlled interference environment, a Faraday cage is used to keep all uncontrolled interference from entering the test setup. To avoid multipath fading, the chamber walls and ground are covered with absorbing materials. To generate the controlled interference, a Raspberry Pi 3 (RPi 3) is used to generate Wi-Fi interference. The possible Wi-Fi channels used are channels 1, 6 or 11, and the Wi-Fi transmission power is set to 30 dBm. Furthermore, JamLab-NG is used to control the Wi-Fi interference [29]. To show the influence of the Wi-Fi interference from the RPi 3, the nRF boards are located and fixed, on a wooden table, on the opposite sides of the RPi 3. The distance between the two development boards is approximately 90 cm. The experimental setup is shown in Figure 6a.

#### 6.1.2. Uncontrolled Environment

To evaluate the proposed IAS and CSA in an uncontrolled environment, both nRF boards are placed in an office environment. In the office environment, multiple Wi-Fi channels are used, as well as functioning bluetooth and BLE devices. They are placed on a desk having a distance of 30 cm between them. Two laptops are placed near the two nRF boards, respectively, to generate Wi-Fi interference on Wi-Fi channels 1 and 10. The experimental setup is shown in Figure 6b.

### 6.2. Channel Quality Baseline

**Figure 7:**
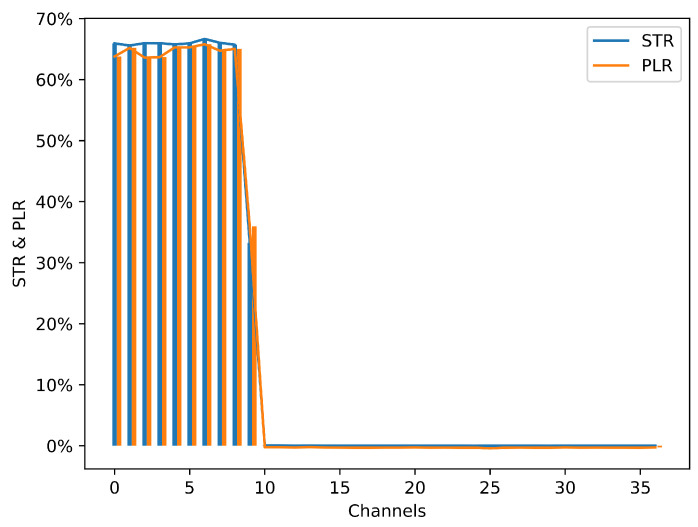
STR and PLR under interference from RPi 3 (10,000 connection events are tested for each channel).

In the first experiment, using the controlled environment, a baseline channel quality is determined. Each BLE channel, from 0 to 36, is tested for 10,000 connection events. To evaluate the baseline interference from Wi-Fi on BLE, the RPi 3 sends a continuous stream of data on Wi-Fi channel 1. The result of this experiment is shown in Figure 7. It clearly shows that the BLE channels coinciding with Wi-Fi channel 1 are affected while others are not. BLE channels 0 to 8 show a STR and PLR around 65% corresponding to the maximum duty cycle of the Wi-Fi. On BLE channel 9 a lower STR and PLR exist due to the low power at the edge of the Wi-Fi channel. It should be noted that both the STR and the PLR have similar results, which implies that they have similar capabilities to show the interference. Besides that, the STR is a bit higher than PLR indicating that the ST event has a higher chance of detecting the interference, except channel 9.

To confirm the interference environment, the same test is repeated with the RPi 3 completely turned off, resulting in exactly 0% for all BLE Channels. The main conclusion from this baseline experiment is that both ST event and PL event can be used to detect interference. However, both are used in the IAS proposed in this paper to ensure maximum detectability.

This experiment illustrates the impact of Wi-Fi interference on the BLE connection reliability under the experiment setup of Figure 6a. Both the STR and the PLR are applied to quantify the impact, thus giving the corresponding baseline channel quality. For example, the 65% STR and PLR suggest that a packet transmitted on that channel has a probability of 65% to face PL or error, thus the probability of a successful transmission on that channel is only 35%.

### 6.3. Feasibility of IAS in Existing CSAs

As a second experiment, the IAS is implemented in both the nRF52840 DK in combination with CSA #2. A connection is set up in the uncontrolled office environment while the available channels are monitored. The goal of this experiment is to check if the IAS works properly under the official BLE specification and how efficiently the IAS detects the environment.

In the Zephyr RTOS, the channel map is saved as [0xff, 0xff, 0xff, 0xff, 0x1f], a little-endian system. The first 0xff indicates that the first eight channels, 0–7, are available. As shown in Table 2, at the beginning of the connection, all the channels are set as available by default. At connection event 30, the master updates the channel map because there are ST events or PL events happening on a channel. At connection event 102, the IAS has detected all the channels under the interference of Wi-Fi channel 1. If the CI is set as 7.5 ms, all the channels under Wi-Fi channel 1 interference are detected and blocked after approximately 765 ms. After that, the channel map keeps being updated again when the IAS detects other interference.

In Table 2, a fatal phenomenon is shown, occurring during connection event 59,642 in the experiment. At this point in time, only two channels are left to be used, being channels 10 and 32. Due to the random interference, the number of available channels in the channel map slowly diminished. Since BLE needs at least two channels, a fatal error occurs when one of the last two channels are also blocked [8,9]. This shows that the IAS and BLE specified CSAs are not compatible. Hence, a new CSA was developed.

### 6.4. Experiments and Results of Combining the IAS with the Existing and Improved CSA

In this subsection, the IAS and CSAs are combined and tested in different situations. Since the IAS combined with the existing BLE specified CSAs results in less than two available channels, the IAS is modified to better suit the existing CSAs by enabling all channels again after every 400 CIs. This IAS is further referred to as the periodic IAS. Note that this periodic IAS is principally very similar to the implementation proposed in [24]. The difference is Spörk et al. [24] enabled all channels after the number of available ones drops to a specified minimum number. This strategy is achievable but not used because it is easy to cheat after knowing the environment. Therefore, the periodic IAS is used here, enabling all channels after every 400 CIs. We consider the number 400 as a fair choice which avoids enabling all channels too frequently or rarely.

#### 6.4.1. Controlled Fixed Interference

**Figure 8:**
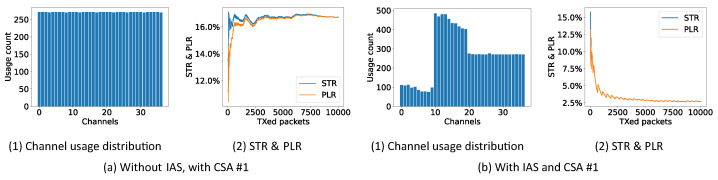
Channel usage distribution, STR and PLR of CSA #1 (10,000 connection events are test for each experiment).

**Figure 9:**
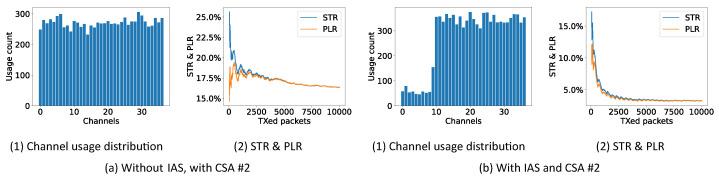
Channel usage distribution, STR and PLR of CSA #2 (10,000 connection events are test for each experiment).

**Figure 10:**
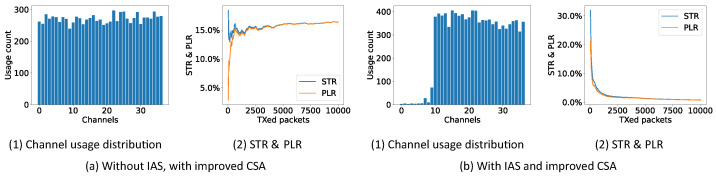
Channel usage distribution, STR and PLR of improved CSA (10,000 connection events are test for each experiment).

In the first experiment, CSA #1, CSA #2 and the improved CSA are tested with or without the (periodic) IAS. The experiment is conducted in the controlled environment where the RPi 3 sends a continuous stream of data on Wi-Fi channel 1. In the experiment, the master initiates 10,000 connection events to evaluate the performance. Figure 8, Figure 9 and Figure 10 show the performance results in the form of the STR and PLR. They also show the channel usage distribution. The PLR is used as the main performance indicator as it has been commonly used in literatures [24,25,30]. The STR is included to see the ST and CRC status.

In Figure 8a, it is shown that the channel usage distribution of CSA #1 is uniform over all channels, as mentioned in [31,32]. After 5000 CI, the STR and PLR converge to approximately 17% close to the theoretical value of 16.7% (65% Wi-Fi duty cycle ·$\frac{9.5}{37}$ Wi-Fi channel occupancy = 16.7% ). If the periodic IAS is used together with CSA #1, as shown in Figure 8b, the channel usage distribution shows a clear drop at the channels which are used by the Wi-Fi channel. The STR and PLR now drop to 2.73% showing that simply using the periodic IAS together with CSA #1 is already a huge improvement. The periodic zigzag in the STR and PLR originates from the periodic IAS. When the STR and PLR increase, all channels have been enabled. After the increase, the IAS blocks the Wi-Fi channels again leading to a decrease in STR and PLR.

In Figure 9a, it is shown that the channel usage distribution of CSA #2 is again uniform but a bit more rough over all channels as mentioned in [31,32]. Again, the STR and PLR converge to approximately 16.5% close to the theoretical value of 16.7%. If the periodic IAS is used together with CSA #2, as shown in Figure 9b, the channel usage distribution shows a clear drop at the channels which are used by the Wi-Fi channel and a more uniform distribution than CSA #1 over the remaining channels. The STR and PLR drop to 3.26% again showing the performance of the periodic IAS.

Figure 10a shows that the improved CSA without the IAS does not seem to improve on any parameter. It results in an equal performance as the other CSAs. However, when combining the new improved CSA with the regular IAS, as shown in Figure 10b, the performance clearly improves. The STR and PLR drop to 0.78% and 0.74%, respectively. The distribution shows that channels 0–10 have a very low, but not zero, usage, due to the fact that the improved CSA never really blacklists a BLE channel. It only reduces the possibility to use it, drastically.

In all three cases, the STR and PLR differ at the beginning of the experiment and converge at the end. The reason is that the number of connection events is too low at the beginning. Thus, even a small difference, between the numbers of ST events and PL events, will lead to a huge divergence of the ratios.

#### 6.4.2. Controlled Random Interference

In the second experiment, a more harsh and dynamic environment is created in the semi-anechoic chamber. The RPi 3 is set to generate Wi-Fi interference on different Wi-Fi channels 1, 6 or 11, randomly. The duration of the interference at a selected Wi-Fi channel is also random, between 1 and 10 s approximately. Under this kind of environment, the IAS and improved CSA are pushed to their performance limits. To find the limits and be sure of that the results converge, the number of connection events is set to 100,000.

**Figure 11:**
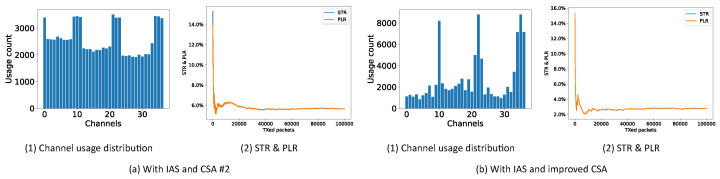
STR and PLR of CSA #2 and the new improved CSA in a controlled random environment.

Figure 11 shows the performance of CSA #2 together with the periodic IAS and the new improved CSA with the regular IAS. CSA #1 is not considered in this test since mainly CSA #2 is recommended. It can be seen that, even at the beginning of the communication in this random and dynamic environment, both solutions do a good job in bringing down the PLR in comparison with the expected 16.7% PLR. However, the improved CSA results in a 2.8% PLR after 100,000 packets, while CSA #2 only reaches 5.65%.

#### 6.4.3. Uncontrolled Interference

**Figure 12:**
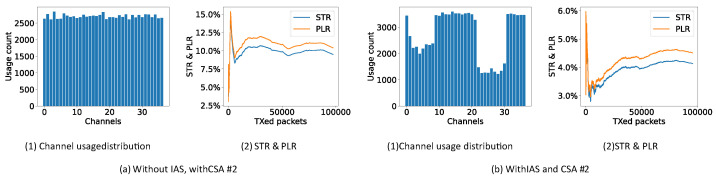
Channel usage distribution, STR and PLR of CSA #2 with IAS in an uncontrolled environment.

**Figure 13:**
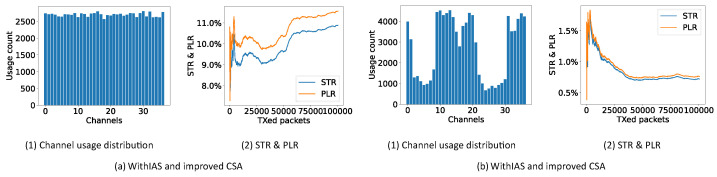
Channel usage distribution, STR and PLR of improved CSA with IAS in an uncontrolled environment.

As a final experiment, both CSA #2 together with the periodic IAS and the improved CSA with the regular IAS are tested in the uncontrolled interference environment (an office environment). In this experiment, the nRF boards are placed on a desk with a distance of 30 cm between them. Two laptops are placed near the two nRF boards while both of them are using Wi-Fi to play some 4K live videos using the Wi-Fi channels 1 and 10 simultaneously. In Figure 12, the STR and PLR values are shown with and without the IAS. From these results, we can again confirm that the periodic IAS with CSA #2 has only half of the PLR as without the periodic IAS. In the channel usage distribution, it can be seen that we indeed have two Wi-Fi channels at channel 1 and 10. In Figure 13, the same is shown, now using the improved CSA. In this case the PLR drops even lower to approximately 0.8%. There are a few reasons for the drop from 2.8% to 0.8%. Firstly, the distance and position of BLE and Wi-Fi changed. Secondly, the pattern of Wi-Fi interference changed from the JamLab-NG one to the laptop ones. Lastly, from our other equipment, we saw that our laptops did not occupy Wi-Fi channels all the time, even when playing 4K live videos. It should be noted that in this test, another unknown and uncontrolled interference existed around BLE channel 16, again confirming the effectiveness of the proposed improved CSA.

#### 6.4.4. Summary of the Experiments

The results of all experiments are combined in Table 3 showing all PLR and STR values after 10,000 or 100,000 connection events. In every experiment, the new improved CSA reaches lower PLR and STR values and even reaches a PLR of 0.8% in an uncontrolled and only partly determined environment, proving its effectiveness. In all cases, be it random, fixed or uncontrolled, the PLR is always lower than 3.0%, which is considered as reliable wireless link [30,33]. The results in [24] are comparable to the fixed controlled Wi-Fi interference test using CSA #1 and CSA #2. With their implementation of enabling all channels when a certain limit of unavailable channels is reached, they reach a value of approximately 1.4% PLR, which is still approximately double of the new improved CSA.

#### 6.4.5. Discussion for IAS and Improved CSA

Our results provide users and developers an idea about the BLE connection robustness under interference, especially under Wi-Fi interference. The developers of BLE may implement our methodology or a part of it into their products according to the application or environment. Moreover, the BLE special interest group who defines and updates the BLE specifications may integrate our methodology into BLE standards for future usage if possible.

Both the IAS and the improved CSA are tested under different harsh environments and proved for their performance under interference. However, due to its complexity, there is no need to implement them in an interference-free environment. They are more recommended in harsh dynamic environments, such as office and industry, or critical situations, such as hospitals. Our experiments show the performance limitations of the IAS and the improved CSA mainly under Wi-Fi interference. To further explore their limitations, some even harsher cases could be considered, e.g., more Wi-Fi interference, multiple BLE pairs and other interference in the 2.4 GHz band. What is certain is that BLE devices will try to avoid all the interference they meet. The worst case is all the channels are with very low availability, which will lead to a uniform channel usage distribution. However, to know the details, more investigation in these situations is needed.

## 7. Conclusions

The robustness of wireless communication, including BLE, is crucial for successful deployments in reality. Electromagnetic interference (EMI) could severely impact the performance of BLE. Therefore, it urges reasonable and effective solutions.

For this purpose, three contributions are presented in this paper: first, an IAS is derived to detect and monitor interference and communication quality at the link layer level; second, an improved CSA is proposed to avoid interference, by sampling and learning from environment; and, third, the IAS and improved CSA are combined and tested under extensive experiments on real BLE devices to characterize the performance of such a mechanism.

The influence of the IAS and improved CSA on BLE communication robustness is evaluated in this paper. The IAS can become aware of interference quickly, continuously and accurately using link-layer connection events. The improved CSA raises the availability from one channel map to multiple ones. The performance of the IAS and improved CSA are evaluated by three parameters or metrics: the channel usage distribution, STR and PLR. Experimental results clearly illustrate the impact of IAS and improved CSA: both STR and PLR are less than 3.0% in the chamber and 1.0% in the office. Besides, this would be a full set of link layer solutions for BLE connection robustness.

Regarding the possibility of future work, we envision extending the investigation to other directions. Some potential side products from the IAS and improved CSA can be analyzed theoretically and tested as follows. First, the IAS and improved CSA are able to detect interference around both sides of BLE devices, because they rely on the BLE connection status. Second, since both the IAS and improved CSA depend on the interference environment, which is totally random, the randomness and anti-eavesdropping could be improved. Besides, both improvements are located in the BLE link layer, which implies the universality for all the BLE devices.

## Figures and Tables

**Table 1:** The parameters used for the test bed.

Parameters	Values
Hardware	nRF52840 DK
Software	Zephyr RTOS
Connection interval (CI)	7.5 ms (30–50 ms by default, then updated to 7.5 ms)
Connection latency	0
Supervision timeout (ST)	4 s
Maximum transmission unit	23 bytes
PHY mode	LE 1M PHY

**Table 2:** The channel map updating speed while using CSA #2 from official BLE specification.

Connection Event	Channel Map	Number of Available Channels
0	[0xff, 0xff, 0xff, 0xff, 0x1f]	37
30	[0xff, 0xff, 0xff, 0xdf, 0x1f]	36
38	[0xfb, 0xff, 0xff, 0xdf, 0x1f]	35
55	[0xbb, 0xff, 0xff, 0xdf, 0x1f]	34
59	[0xbb, 0xfe, 0xff, 0xdf, 0x1f]	33
62	[0xb9, 0xfe, 0xff, 0xdf, 0x1f]	32
69	[0x39, 0xfe, 0xff, 0xdf, 0x1f]	31
73	[0x29, 0xfe, 0xff, 0xdf, 0x1f]	30
83	[0x21, 0xfe, 0xff, 0xdf, 0x1f]	29
96	[0x20, 0xfe, 0xff, 0xdf, 0x1f]	28
102	[0x00, 0xfe, 0xff, 0xdf, 0x1f]	27
……	[……]	……
59642	[0x00, 0x04, 0x00, 0x00, 0x01]	2

**Table 3:** The results of STR and PLR under different conditions (the * represents no data tested for that condition).

		CSA #1	CSA #2	Improved CSA
fixed controlled Wi-Fi interference (after 10,000 connection events)	STR	2.73%	3.27%	0.78%
	PLR	2.73%	3.26%	0.74%
random controlled Wi-Fi interference (after 100,000 connection events)	STR	*	5.65%	2.78%
	PLR	*	5.65%	2.80%
uncontrolled Wi-Fi interference (after 100,000 connection events)	STR	*	4.51%	0.76%
	PLR	*	4.13%	0.72%
